# The Radiation Therapy Technology Evidence Matrix: a framework to visualize evidence development for innovations in radiation therapy

**DOI:** 10.3389/fonc.2024.1351610

**Published:** 2024-04-02

**Authors:** Sarah Edwards, Marco Luzzara, Veronica Dell’Acqua, John Christodouleas

**Affiliations:** Medical Affairs and Clinical Research, Elekta AB, Stockholm, Sweden

**Keywords:** adaptive, clinical evidence development, Evidence Matrix, innovation, framework, radiation therapy, technology

## Abstract

Clinical evidence is crucial in enabling the judicious adoption of technological innovations in radiation therapy (RT). Pharmaceutical evidence development frameworks are not useful for understanding how technical advances are maturing. In this paper, we introduce a new framework, the Radiation Therapy Technology Evidence Matrix (rtTEM), that helps visualize how the clinical evidence supporting new technologies is developing. The matrix is a unique 2D model based on the R-IDEAL clinical evaluation framework. It can be applied to clinical hypothesis testing trials, as well as publications reporting clinical treatment. We present the rtTEM and illustrate its application, using emerging and mature RT technologies as examples. The model breaks down the type of claim along the vertical axis and the strength of the evidence for that claim on the horizontal axis, both of which are inherent in clinical hypothesis testing. This simplified view allows for stakeholders to understand where the evidence is and where it is heading. Ultimately, the value of an innovation is typically demonstrated through superiority studies, which we have divided into three key categories – administrative, toxicity and control, to enable more detailed visibility of evidence development in that claim area. We propose the rtTEM can be used to track evidence development for new interventions in RT. We believe it will enable researchers and sponsors to identify gaps in evidence and to further direct evidence development. Thus, by highlighting evidence looked for by key policy decision makers, the rtTEM will support wider, timely patient access to high value technological advances.

## Introduction

Clinical hypothesis testing of new radiation therapy (RT) technologies and treatment techniques is vital to ensure the safe and effective delivery of cancer care. There needs to be progression through the testing stages to demonstrate the value and potential superiority of innovations as outlined by the R-IDEAL clinical evaluation framework (1).

Research using these new technologies and techniques, naturally occurs across the globe with key academic centers often at the forefront of clinical testing hypothesis. With the research ongoing at a global level, how do we know that the evidence for a new technology is progressing sufficiently to demonstrate its value and to enable wider patient access to an innovative treatment technique?

Existing evidence classification models such as the Evidence Pyramid (2) and Grades of Recommendation, Assessment, Development and Evaluation (GRADE) (3)are mono-dimensional.

In short, we need a method that shows:

• How evidence for an innovation in radiation therapy is developing
• How the gathering evidence relates to authorities’ need for data to allow access for patients.

The method should make this information quickly clear to a variety of stakeholders, particularly those who may not be familiar with clinical research terminology.

Our Radiation Therapy Technology Evidence Matrix (rtTEM) uniquely helps visualize the evidence status and the gaps, giving a deeper understanding of, and the importance of, what is going on, thereby giving stakeholders confidence that evidence is both being developed and becoming available to eventually support wider patient access.

## Materials and methods

We created a two-dimensional matrix based on the R-IDEAL clinical hypothesis testing stages (1), which is illustrated in **Supplementary Figure S1**. We then adapted the R-IDEAL framework to allow for visualization along two axes in hypothesis testing, namely the type of the claim (the stage along the R-IDEAL journey) and the strength of the evidence. The strength rating is consistent with evidence levels already used in the medical community (4), with the inclusion of the size of the patient cohort. The enrolment criteria were established based on clinical judgment by a group of subject matter experts, drawing upon their knowledge of clinical trial design. These criteria were intended to illustrate the broader evidence generation for a Radiation Therapy technology and not limited to specific clinical conditions. They ensure consistency, and enable automation in analysis, while reflecting progression in the type of the claim and the strength of the evidence.

**Figure 1:**
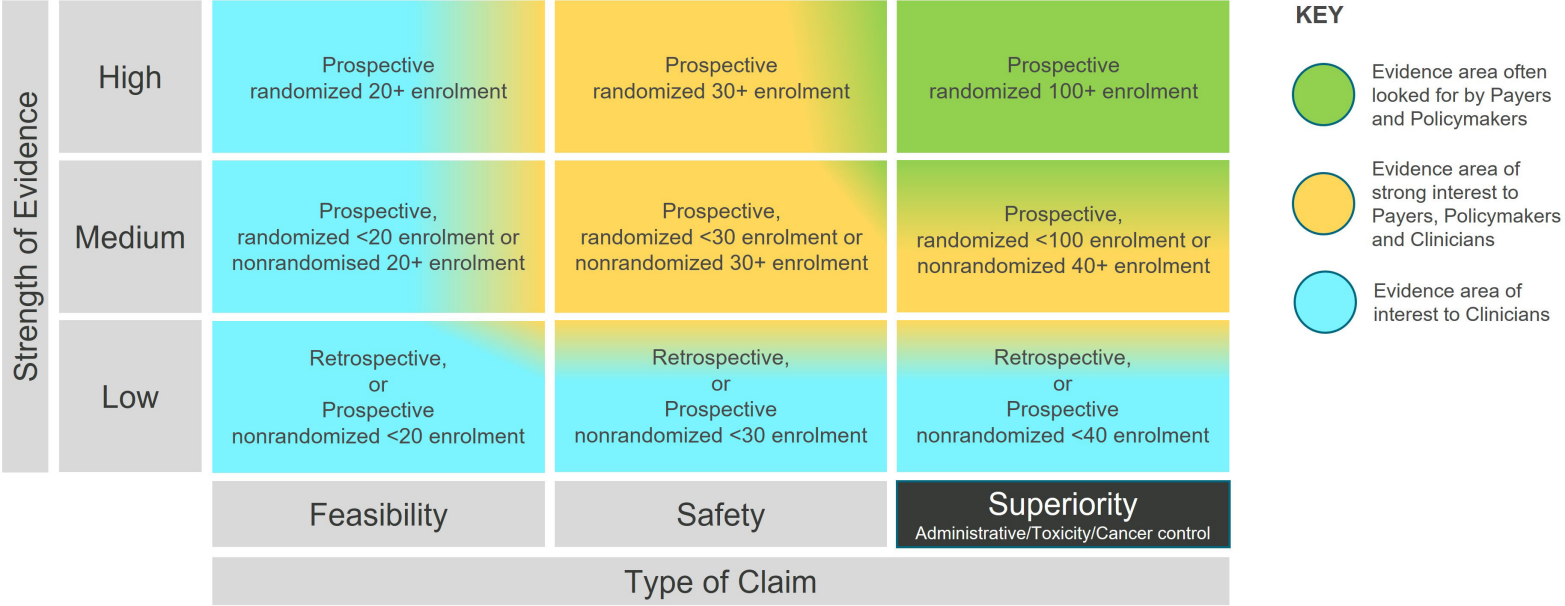
The Radiation Therapy Technology Evidence Matrix (rtTEM).

The rtTEM displayed in **Figure 1** follows a 3 by 3 arrangement, which serves as a standard layout for easy visualization and comparison. This format is particularly useful for new technologies with limited evidence, preventing a cluttered matrix with excessive white space. Studies in the superiority stage can be further classified under one of three categories: administrative, toxicity, and cancer control, noting that non-inferiority studies may presume superiority in one of the above and therefore be classified as superiority. While maintaining the 3x3 display format, such additional granularity can be incorporated as discussed in the results. This concise arrangement facilitates clear understanding and comparison of evidence development.

To illustrate use of the rtTEM, the publication ‘Stereotactic ablative radiation for pancreatic cancer on a 1.5 Tesla magnetic resonance-linac system’ (5) reports on 30 patients that were treated with ablative radiation therapy using online magnetic resonance guided adaptive radiation therapy (MRgART). The rtTEM classifies this as a medium strength, safety article. The MIRAGE study (clinicaltrials.gov NCT04384770) (6) is a prospective, randomized trial with an enrollment of 179 patients. The rtTEM classifies MIRAGE as a high strength, superiority trial. These examples can be visualized on the rtTEM in **Supplementary Figure S2.**

We used online and real-time MRgART evidence development as an example of a current technical innovation in radiation therapy. We used online cone beam computerized tomography guided adaptive radiation therapy (CBCTgART) as an example of an emerging technology, and proton beam therapy (PBT) and intensity modulated radiation therapy (IMRT) as examples of mature technologies. It should be noted that the purpose of the analysis conducted for PBT and IMRT, where there is extensive evidence, was to demonstrate the matrix methodology and not intended as a definitive analysis on the status of the technology itself.

The trials were identified in Clinicaltrials.gov and publications were collected by monitoring peer reviewed journals. Further details of data selection are given in **Supplementary Data**, and search terms used are listed in **Supplementary Table S1.**

## Results

The rtTEM (**Figure 1**) highlights that for radiation therapy technologies to have a lasting impact, evidence is needed in the top right-hand area (green shading) as this is the evidence often looked for by high-level decision makers, e.g., reimbursement and healthcare policy makers, who have significant influence over market access to new interventions. Once clinical safety and efficacy have been demonstrated, evidence of superiority helps to answer questions, such as: Why invest in an innovation compared to what is currently available?

## The rtTEM can be used to visualize the evidence status for a radiation therapy technology at a point in time

An analysis of publications shows actual evidence from clinical studies, while an analysis of open trials shows the potential evidence being developed. Together these allow us to fully visualize the evidence generation journey toward improved patient access. This is illustrated by using online and real time MRgART clinical evidence.

**Figure 2:**
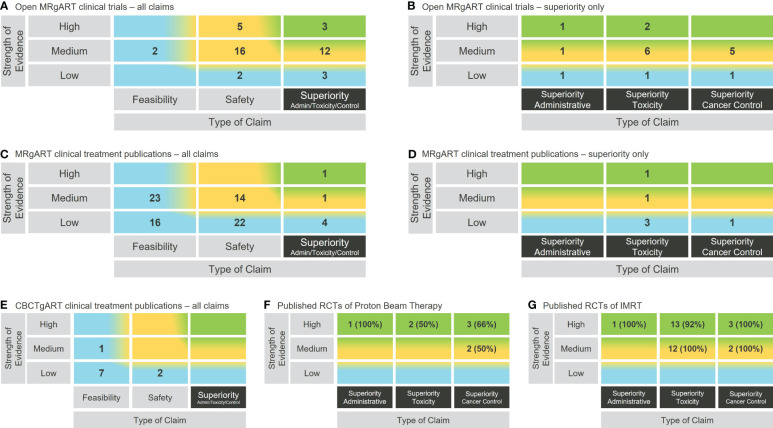
Use of the rtTEM to visualize the clinical evidence status for example RT technologies. **(A)** Open clinical trials analysis for online and real-time MRgART. **(B)** Zoomed in view on the open online and real-time MRgART clinical trials superiority claims. **(C)** Clinical treatment publications analysis for online and real-time MRgART. **(D)** Zoomed in view on the online and real-time MRgART clinical treatment publications superiority claims. **(E)** Clinical treatment publication analysis for online CBCTgART. **(F)** Zoomed in superiority claim analysis of published RCTs on PBT (% of publications that support trial primary endpoint). **(G)** Zoomed in superiority claim analysis of published RCTs on IMRT (% of publications that support trial primary endpoint).

At the end of November 2023, there were 43 open clinical trials listed on ClinicalTrials.gov designated R-IDEAL stage 2 or above (**Supplementary Table S2**) and 81 clinical treatment publications (**Supplementary Table S3**) on either ViewRay MRIdian or Elekta Unity MR guided Linacs. These were assessed and plotted on the rtTEM, and the results shown in **Figures 2A–D**.

## The rtTEM can be used to zoom in or zoom out to visualize evidence at difference levels of granularity

Different levels of granularity are illustrated in **Figures 2A–D**, where both an overall zoomed out view, and a zoomed in view on the superiority claims, of the clinical evidence for online and real-time MRgART is demonstrated using the same matrix structure.

## The rtTEM can be used to compare the clinical evidence status of different technologies

The rtTEM is applicable to all RT technologies and can therefore be used to analyze their evidence status and consequently compare between competing technologies. This is demonstrated in **Figures 2E–G**, where the rtTEM is applied to online CBCTgART, as an example of another emerging technology in comparison with MRgART, and to PBT and IMRT, as examples of established technologies. (**Supplementary Tables S4**–**S6** list the publications used.) For mature technologies it is appropriate to focus on the higher-level evidence status than the lower-level evidence build up happening for emerging technologies. We therefore illustrate a deeper level of granularity by zooming in on the evidence from randomized control trials (RCTs) for PBT and IMRT– i.e., the evidence area toward the top right-hand corner of the rtTEM. In this zoomed in visualization (**Figures 2F, G**), the addition of the percentage information provides an immediate insight into the number of publications reporting on RCTs that were supportive (i.e., the primary endpoint of the trial was demonstrated) of each of the example mature technologies.

## Discussion

That there is a need for standardization in evidence appraisal for innovation in radiation therapy has been highlighted by the European Network for Health Technology Assessment (EUnetHTA) project which seeks to create an effective and sustainable network for health technology assessment across Europe (7). The rtTEM supports this standardization, serving as a visual representation of the evidence landscape for innovations in radiation therapy.

We demonstrated in **Figure 2** that the rtTEM can be used to visualize the clinical evidence status for a RT technology as a snapshot in time and over time. **Figures 2A–D** also shows that there has already been a lot of work establishing safety and feasibility of MRgART, and the community is now focusing on assessment of superiority. This indicates that over time the superiority trials will lead to superiority publications though they will not necessarily support the hypothesis of superiority. The growth of evidence availability in the top right-hand area (green shading) illustrates the evidence development journey through the R-IDEAL testing stages and progress over time.

**Figure 2** also illustrates the rtTEM can be used to zoom in on selected superiority evidence. The zoomed in view on the superiority column in **Figures 2B, D**, provides a detailed breakdown of the types of claims being studied and reported on, for online and real-time MRgART. Similarly, **Figures 2F, G** demonstrates a focus on the higher-level superiority studies reported on by analyzing RCT publications only, for two mature RT technologies, PBT and IMRT. These RT technologies have been available for many years and would therefore be expected to have such higher level of evidence.

We demonstrated in **Figure 2** that the rtTEM can also be used to compare evidence for different technologies using the published clinical treatment results for two emerging RT technologies namely online CBCTgART and MRgART, and the published RCTs for two mature technologies, namely PBT and IMRT. Online CBCTgART is seen to have far fewer, and less strength evidence available (**Figure 2E**). This is to be expected as online CBCTgART started clinical use in 2020, whereas MRgART started in 2014. Over time the reported evidence would be expected to increase for both. The comparison for PBT and IMRT zooms in on RCTs as a higher-level evidence example (**Figures 2F, G**). At this depth of analysis, the inclusion of the percentage of supportive publications (i.e., those that demonstrate the primary endpoint of the trial) is a useful insight when considering wider patient access to a technology by high level decision makers and for researchers seeking to address the evidence gap.

In contrast to existing evidence classification models, the rtTEM provides a unique 2D visualization of the status of the evidence for a particular innovation by showing both the hierarchy and the strength of the evidence. The Evidence Pyramid and any derived models, focus on the design of the study and indicate very well the evidence hierarchy that exists while recognizing that the quality of evidence is not so easily visualized (2). Quality analysis is comprehensively addressed by the Grades of Recommendation, Assessment, Development, and Evaluation (GRADE) methodology. GRADE provides a system for rating quality of evidence and grading the strength of recommendations to inform healthcare decisions by a variety of stakeholders including clinicians and policymakers (3). In comparison to both these established models, the rtTEM provides a simplified 2D view of the type and potential strength of the evidence being developed or reported on. In addition to demonstrating progress and identifying gaps in evidence for researchers, we believe that such a clear visualization will also help financial and medical policy decision makers to evaluate the development of evidence for MedTech innovations, giving them confidence that initial hypotheses and findings are correct and continue to be built on (8). We therefore propose it could be used within an RT value-based assessment framework, such as that being developed by the ESTRO-HERO project (9). This project highlights the lack of common definitions for radiation therapy innovations and is planning to define criteria for an RT specific value-based assessment tool. The rtTEM could be valuable in demonstrating the evidence requirements for differently defined RT innovations within that tool.

That the rtTEM can be applied to all technological innovations in RT is shown by using online and real-time MRgART, online CBCTgART, PBT and IMRT in this paper. We believe it can potentially be used for other medical technologies that incrementally develop use of a single medicine, for example focused ultrasound (10) or robotic surgery (11). We do not propose its use for pharmacological innovations where evidence is developed before release of the product. The key purpose of the rtTEM is to see the evidence status and gaps.

The rtTEM has been designed to provide a generic overview of the evidence generation. As a simplified model there are limitations in what it shows and can be used for.

A key limitation is that the strength of evidence is based on the study design and the number of patients. Those two factors alone may result in an incorrect determination of this strength. For very novel innovations (e.g., online PET guided RT) a deeper analysis of the effect/endpoints under evaluation and the statistical strength of the results might be more appropriate. However, at this first introduction, the use of number of patients means the model is easily reproducible and doesn’t require an expert (potentially subjective) review of each single paper/trial. The scope of this implementation of the rtTEM is to provide an enlightened starting point for a more in-depth analysis.

Despite striving for simplicity, some expertise is required when considering the superiority studies highlighted by the rtTEM. The model does not identify superiority compared to what, and the detail of the studies need to be looked at to determine this and to ensure a relevant standard of care comparator has been chosen.

These are important factors for key stakeholders such as policy makers to consider when using the rtTEM. The model is designed to illustrate when there is potentially enough evidence to enable a Health Technology Assessment or QALY analysis to take place, where further in-depth analysis of each publication and trial will be required (12) (13).

Also, the rtTEM user likely needs to have the context about what to expect when visualizing the status of a single technology, For example, online CBCTgART began clinical use in 2020 and is therefore at an early stage of clinical evidence development as can be seen from the lower level of evidence available in contrast with the higher level of evidence zoomed in on for PBT and IMRT, both of which have been in clinical use for over 20 years (**Figures 2E–G**). That online CBCTgART has limited evidence is not a reflection of its clinical applicability or usefulness but an outcome of its stage in evidence development. Therefore, the need for some expertise in the technology area is required as it would be when looking at any summary information.

Another limitation is that the matrix outcomes can be sensitive to the evidence requirements applied. For example, in **Figures 2A–D**, we included only clinical hypothesis testing trials for online and real-time MRgART that we designated R-IDEAL stage 2 and above, and clinical treatment publications that reported on more than 5 patients (**Supplementary Data Section 1**.) This means that without looking at the details, it is not easy for a reader to know what criteria have been applied. As experience develops with the utilization of the rtTEM, we expect that “standard” criteria will emerge in the same way that they have emerged for other ways of summarizing complex information, such as disease-free survival and progression free survival when showing these outcomes in a Kaplan Meier curve.

In conclusion, a new model - the rtTEM - for visualizing and monitoring evidence development for RT technical innovations has been described. This model has been shown to be a flexible way of visualizing both on-going clinical studies as well as clinical trial publications. The model can be used to visualize the maturation of technologies over time as well as compare and contrast the maturity of technologies at a given point in time. We propose that the rtTEM is of value to efficiently monitoring the development of evidence for any RT technique. We anticipate its wider utilization to allow a greater understanding of evidence generation and to ensure that the status of evidence development for innovations is clearly visible to all stakeholders.

## Data availability statement

The original contributions presented in the study are included in the article/**Supplementary Material.** Further inquiries can be directed to the corresponding author.

## Author contributions

SE: Writing – original draft, Conceptualization, Investigation, Methodology, Visualization. ML: Conceptualization, Investigation, Methodology, Writing – review & editing. VD: Investigation, Methodology, Writing – review & editing. JC: Conceptualization, Methodology, Writing – review & editing.
