# Supplementary material for: The Radiation Therapy Technology Evidence Matrix: a framework to visualize evidence development for innovations in radiation therapy
Source: Front Oncol. 2024 Apr 2;14:1351610. doi: 10.3389/fonc.2024.1351610 (PMC11018969; doi:10.3389/fonc.2024.1351610)
Supplement: Supplementary file 1 [file DataSheet_1.pdf]

Supplementary Material

1 Supplementary Data

1.1 MRgART data selection

We defined online MRgART as using the information coming from an MR scan while the patient is on the treatment table to either ensure that the original treatment plan is delivered as planned, or to modify the treatment plan based on the new information. We defined real-time MRgART as the ability to automatically adapt the treatment plan and delivery to the changing patient anatomy during the treatment delivery. This is in accordance with the ICRU Report 97: MRI-Guided Radiation Therapy using MRI-Linear Accelerators.

ClinicalTrials.gov was used as the main source for the clinical trials in the analysis presented here, as this is the most globally used registry. Other clinical trials investigating MRgART are known to be open on country databases but have been excluded from this analysis as these are harder to monitor accurately.

Open studies were defined as those with recruitment status: not yet recruiting, recruiting, active not recruiting, or enrolling by invitation. The filtered results were then reviewed, online and real-time MRgART studies were selected and assigned a type: clinical, registry or technical. Clinical studies are defined as those with a primary outcome that demonstrates clinical outcomes in a specific disease (e.g., HERMES (clinicaltrials.gov NCT04595019), registries have large enrolment numbers across multiple diseases (e.g., MOMENTUM (clinicaltrials.gov NCT04075305) and technical studies are those that are seeking to prove a technical endpoint such as ability to treat a patient on the new technology in a certain time frame (e.g., PRISM (clinicaltrials.gov NCT04595019).

We selected studies designated R-IDEAL stages 2a, 2b and 3 (feasibility/safety and superiority), as these are the most important initially to support expansion of patient access to a new intervention.

Clinical treatment publications used in the analysis were done on commercially available MR-Linac systems and reported on more than 5 patients as these represent more robust clinical outcomes.

1.2 CBCTgART data selection

The clinical treatment publications were collected by monitoring peer reviewed journals for online CBCTgART, where online is defined as using the information coming from the CBCT imaging while the patient is on the treatment table to either ensure that the original treatment plan is delivered as planned, or to modify the treatment plan based on the new information.

Clinical treatment publications used in the analysis were done on commercially available online CBCTgART systems and reported on more than 5 patients as these represent more robust clinical outcomes.

Restricted Information and Basic Personal Data

1.3 PBT and IMRT data selection

We collected randomized control trial publications for PBT and for IMRT. Publications for protons were accepted where the comparison was to conventional treatment. Publications for IMRT, including volumetric modulated arc therapy (VMAT), were accepted where the comparison was to a standard of care using a different technology (e.g., 3D conformal treatment). Studies exploring IMRT using different dose regimes or where multiple technologies were being used (e.g., IMRT and image guided radiation therapy (IGRT)) were rejected. For both PBT and IMRT any interim results were removed. The remaining studies were assessed for the type of superiority claim as well as whether the paper was supportive or not of the technology for the claim under investigation.

2 Supplementary Figures and Tables

2.1 Supplementary Figures

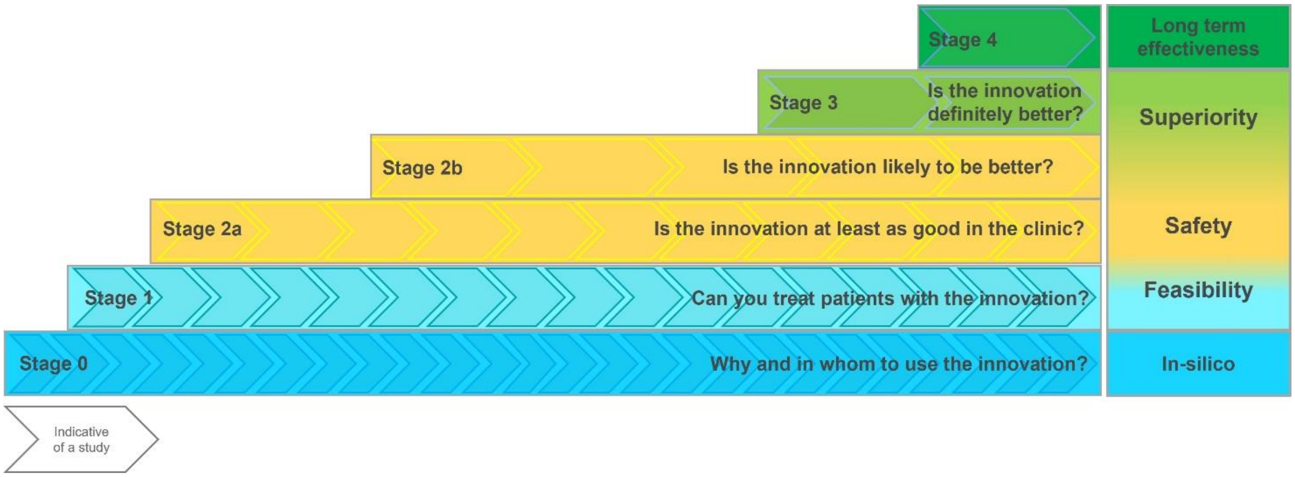

Supplementary Figure S1. Stages of Clinical Hypothesis Testing as defined by the R-IDEAL framework.

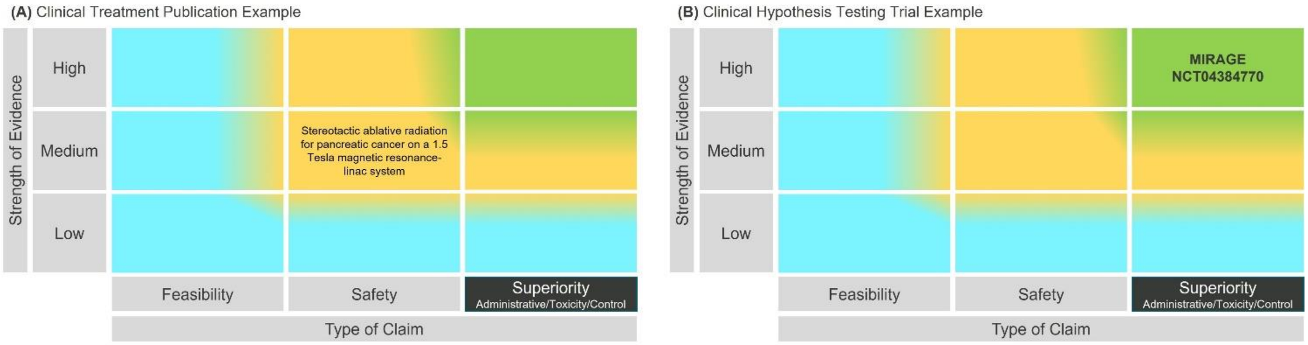

Supplementary Figure S2: Example of **(A)** a clinical treatment publication and **(B)** a clinical hypothesis testing trial plotted on the rtTEM.

Restricted Information and Basic Personal Data

2.2 Supplementary Tables

Table S1: Search Terms Used

Technology	Database	Search terms	Date of last search
MRgART	Clinicaltrials.gov	Cancer, ((MR OR MRI) AND guided AND radiation therapy) NOT Ultrasound	30th November 2023
	PubMed	"Elekta"[All Fields] AND "Unity"[All Fields]) OR ("MR-Linac"[All Fields] OR "MRI-guidance"[All Fields]	30th November 2023 *Note: articles were included with future publication dates*
	Google Scholar	elekta OR nucletron<br>MR guided radiotherapy<br>"MRI Linac" OR "MR Linac"	30th November 2023 *Note: articles were included with future publication dates*
CBCTgART	PubMed	((Adaptive[Title/Abstract] AND radiotherapy[Title/Abstract]) NOT MR[Title]) NOT brachytherapy[Title] NOT proton[Title] NOT carbon[Title]	30th November 2023
PBT	PubMed	Radiation AND (proton randomized trial[Title/Abstract])	8th February 2023
IMRT	PubMed	(IMRT[Title/Abstract] OR VMAT[Title/Abstract]) randomized[Title/Abstract] (study[Title/Abstract] OR trial[Title/Abstract]).	15th February 2023

Table S2: Open MRgART clinical trials R-IDEAL stage 2 and above on clinicaltrials.gov

NCT Number	Title	Acronym	Status	URL
NCT05354596	A Multicenter Phase II Study of Stereotactic Radiotherapy for Centrally Located Lung Tumors (STRICT-LUNG STUDY) and Ultra-centrally Located Lung Tumors (STAR-LUNG STUDY).	STRICTSTAR LUNG	Recruiting	https://ClinicalTrials.gov/show/NCT05354596
NCT04997018	A Study of MRI-guided High-dose Radiation Therapy in Prostate Cancer		Recruiting	https://ClinicalTrials.gov/show/NCT04997018
NCT04242342	Adaptative MR-Guided Stereotactic Body Radiotherapy of Liver Tumors	RASTAF	Recruiting	https://ClinicalTrials.gov/show/NCT04242342
NCT05373316	Addition of a Focal Boost in External Beam Radiotherapy for Locally Advanced Prostate Cancer by Online Adaptive MR-guided Radiotherapy	AFFIRM	Not yet recruiting	https://ClinicalTrials.gov/show/NCT05373316
NCT05338866	Chemotherapy Combined With High-dose Radiotherapy for Low Rectal Cancer Using MR Guided Linear Accelerator		Enrolling by invitation	https://ClinicalTrials.gov/show/NCT05338866

Restricted Information and Basic Personal Data

NCT Number	Title	Acronym	Status	URL
NCT04384770	CT-guided Stereotactic Body Radiation Therapy and MRI-guided Stereotactic Body Radiation Therapy for Prostate Cancer, MIRAGE Study		Active, not recruiting	https://ClinicalTrials.gov/show/NCT04384770
NCT02945579	Eliminating Surgery or Radiotherapy After Systemic Therapy in Treating Patients With HER2 Positive or Triple Negative Breast Cancer		Recruiting	https://ClinicalTrials.gov/show/NCT02945579
NCT04861194	EREctile Function Preservation for Prostate Cancer Radiation Therapy (ERECT)	ERECT	Recruiting	https://ClinicalTrials.gov/show/NCT04861194
NCT04595019	Hypofractionated Expedited Radiotherapy for Men With localisEd proState Cancer	HERMES	Recruiting	https://ClinicalTrials.gov/show/NCT04595019
NCT04925583	Magnetic Resonance Guided Adaptive Stereotactic Body Radiotherapy for Lung Tumors in Ultracentral Location	MAGELLAN	Recruiting	https://ClinicalTrials.gov/show/NCT04925583
NCT05027711	Magnetic Resonance-guided Adaptive Stereotactic Body Radiotherapy for Hepatic Metastases	MAESTRO	Recruiting	https://ClinicalTrials.gov/show/NCT05027711
NCT03916419	Magnetic Resonance-Guided Hypofractionated Adaptive Radiation Therapy With Concurrent Chemotherapy and Consolidation Durvalumab for Inoperable Stage IIB, IIIA, and Select IIIB and IIIC Non-small Cell Lung Cancer		Active, not recruiting	https://ClinicalTrials.gov/show/NCT03916419
NCT05114213	MR-Guided Adaptive SBRT of Primary Tumor for Pain Control in Metastatic PDAC	MASPAC	Recruiting	https://ClinicalTrials.gov/show/NCT05114213
NCT04162665	MR-guided Pre-operative RT in Gastric Cancer		Recruiting	https://ClinicalTrials.gov/show/NCT04162665
NCT04896801	MR-guided Prostate Stereotactic Body Radiotherapy in Seven Days	Proseven	Recruiting	https://ClinicalTrials.gov/show/NCT04896801
NCT03972072	MRI - Guided Adaptive RadioTHerapy for Reducing XerostomiA in Head and Neck Cancer (MARTHA-trial)	MARTHA	Recruiting	https://ClinicalTrials.gov/show/NCT03972072
NCT04808323	MRI-Guided Adaptive Radiation Therapy for Organ Preservation in Rectal Cancer		Recruiting	https://ClinicalTrials.gov/show/NCT04808323
NCT04809792	MR-Linac for Head and Neck SBRT		Not yet recruiting	https://ClinicalTrials.gov/show/NCT04809792
NCT04946019	MR-Linac Guided Adaptive FSRT for Brain Metastases From Non-small Cell Lung Cancer		Recruiting	https://ClinicalTrials.gov/show/NCT04946019

Restricted Information and Basic Personal Data

NCT Number	Title	Acronym	Status	URL
NCT05163509	MR-linac Guided Adaptive Radiotherapy for Inoperable Mediastinal Tumor		Recruiting	https://ClinicalTrials.gov/show/NCT05163509
NCT05183074	MR-linac Guided Ultra-hypofractionated RT for Prostate Cancer		Recruiting	https://ClinicalTrials.gov/show/NCT05183074
NCT05037461	Precision Radiotherapy Using MR-linac for Pancreatic Neuroendocrine Tumours in MEN1 Patients	PRIME	Recruiting	https://ClinicalTrials.gov/show/NCT05037461
NCT04422132	Randomized Phase II Trial of Salvage Radiotherapy for Prostate Cancer In 4 Weeks v. 2 Weeks		Recruiting	https://ClinicalTrials.gov/show/NCT04422132
NCT04984343	Randomized Trial of Five or Two MRI-Guided Adaptive Radiotherapy Treatments for Prostate Cancer	FORT	Recruiting	https://ClinicalTrials.gov/show/NCT04984343
NCT03541850	Stereotactic Body Radiation Therapy in Treating Patients With Localized Prostate Cancer That Have Undergone Surgery		Active, not recruiting	https://ClinicalTrials.gov/show/NCT03541850
NCT03621644	Stereotactic MRI-guided On-table Adaptive Radiation Therapy (SMART) for Locally Advanced Pancreatic Cancer		Active, not recruiting	https://ClinicalTrials.gov/show/NCT03621644
NCT04845503	Stereotactic MRI-guided Radiation Therapy for Localized prostatE Cancer	SMILE	Recruiting	https://ClinicalTrials.gov/show/NCT04845503
NCT05375708	Systemic Therapy in Combination With Stereotactic Radiotherapy in Patients With Metastatic Colorectal Cancer up to 10 Metastatic Sites	SIRIUS	Not yet recruiting	https://ClinicalTrials.gov/show/NCT05375708
NCT04726397	UNIty-Based MR-Linac Guided AdapTive RadiothErapy for High GraDe Glioma: A Phase 2 Trial	UNITED	Active, not recruiting	https://ClinicalTrials.gov/show/NCT04726397
NCT04909294	Evaluation of the Efficacy and the Safety of a Stereotaxic Prostatic Radiotherapy Delivered With Linac MRI, in Patients With Prostate Adenocarcinoma	STEREO-RML	Recruiting	https://ClinicalTrials.gov/show/NCT04909294
NCT05350722	Single-dose Preoperative Partial Breast Irradiation in Low-risk Breast Cancer Patients	ABLATIVE-2	Recruiting	https://ClinicalTrials.gov/show/NCT05350722
NCT04407897	SOFT- Stereotactic Ablative Radiotherapy of Infra-diaphragmatic Soft Tissue Metastases	SOFT	Active, not recruiting	https://ClinicalTrials.gov/show/NCT04407897
NCT05301283	Habitat Escalated Adaptive Therapy (HEAT), With Neoadjuvant Radiation for Soft Tissue Sarcoma		Recruiting	https://ClinicalTrials.gov/show/NCT05301283
NCT05603078	A Prospective Study of Preoperative Tumor-bed Boost Followed by Oncoplastic Surgery and Adjuvant Whole Breast Radiotherapy for Early Stage Breast Cancer (BIRKIN)	BIRKIN	Recruiting	https://ClinicalTrials.gov/show/NCT05603078

Restricted Information and Basic Personal Data

NCT Number	Title	Acronym	Status	URL
NCT05600400	Improving Sexual Quality of Life - Randomized Trial of Two vs Five MRI Guided SABR Treatments for Prostate Cancer	iSMART	Not yet recruiting	https://ClinicalTrials.gov/show/NCT05600400
NCT05565521	UNITy-BasED MR-Linac Adaptive Simultaneous Integrated Hypofractionated Boost Trial for High Grade Glioma in the Elderly	UNITED2	Recruiting	https://ClinicalTrials.gov/show/NCT05565521
NCT05720078	UNIty-Based MR-Linac Guided Adaptive RadioThErapy for High GraDe Glioma-3	UNITED-3	Recruiting	https://ClinicalTrials.gov/show/NCT05720078
NCT05731791	Trial to Compare MRI-guided Precision Prone Irradiation (PPI) Versus CT-guided Breast Irradiation	PPI	Recruiting	https://ClinicalTrials.gov/show/NCT05731791
NCT05916040	Total Neoadjuvant Treatment of Rectal Cancer by MRI-guided Radiotherapy	TNTRect	Recruiting	https://ClinicalTrials.gov/show/NCT05916040
NCT05709782	Phase II Cohort of Spinal Stereotactic Radiotherapy in Patients Using a MR LINAC		Recruiting	https://ClinicalTrials.gov/show/NCT05709782
NCT05942742	Estimation of Tumor Response With Linac MRI-guided Adaptive Radiotherapy for Locally Advanced Cervical Cancer	Gynecolunity	Recruiting	https://ClinicalTrials.gov/show/NCT05942742
NCT06050707	MR-Adaptive Radiation Therapy for Anal Cancer With EScalated-Treatment in a Risk-Optimized Approach	MAESTRO	Recruiting	https://ClinicalTrials.gov/show/NCT06050707
NCT06130280	A Study of Single Fraction Stereotactic Body Radiation Therapy (SBRT) Guided by Magnetic Resonance Imaging (MRI) in People With Liver Metastasis From Colorectal Cancer		Recruiting	https://ClinicalTrials.gov/show/NCT06130280

Supplementary Table S3: MRgART Clinical Treatment Publications

Title	Authors	DOI	PMID URL
4D-MRI driven MR-guided online adaptive radiotherapy for abdominal stereotactic body radiation therapy on a high field MR-Linac: Implementation and initial clinical experience.	Paulson ES; Ahunbay E; Chen X; Mickevicius NJ; Chen GP; Schultz C; Erickson B; Straza M; Hall WA; Li XA	10.1016/j.ctro.2020.05.002	https://pubmed.ncbi.nlm.nih.gov/32490218
Accumulated bladder wall dose is correlated with patient-reported acute urinary toxicity in prostate cancer patients treated with stereotactic, daily adaptive MR-guided radiotherapy.	Willigenburg T; van der Velden JM; Zachiu C; Teunissen FR; Lagendijk JJW; Raaymakers BW; de Boer JCJ; van der Voort van Zyp JRN	10.1016/j.radonc.2022.04.022	https://pubmed.ncbi.nlm.nih.gov/35489444

Restricted Information and Basic Personal Data

Title	Authors	DOI	PMID URL
MRI-Guided Online Adaptive Stereotactic Body Radiation Therapy of Liver and Pancreas Tumors on an MR-Linac System.	Stanescu T; Shessel A; Carpino-Rocca C; Taylor E; Semeniuk O; Li W; Barry A; Lukovic J; Dawson L; Hosni A	10.3390/cancers14030716	https://pubmed.ncbi.nlm.nih.gov/35158984
The first patient-reported outcomes from the Utrecht Prostate Cohort (UPC): the first platform facilitating 'trials within cohorts' (TwiCs) for the evaluation of interventions for prostate cancer.	Teunissen FR; Willigenburg T; Meijer RP; van Melick HHE; Verkooijen HM; van der Voort van Zyp JRN	10.1007/s00345-022-04092-2	
First clinical experiences with a high field 1.5 T MR linac.	Bertelsen AS; Schytte T; Moller PK; Mahmood F; Riis HL; Gottlieb KL; Agergaard SN; Dysager L; Hansen O; Gornitzka J; Veldhuizen E; ODwyer DB; Christiansen RL; Nielsen M; Jensen HR; Brink C; Bernchou U	10.1080/0284186X.2019.1627417	https://pubmed.ncbi.nlm.nih.gov/31241387
Initial Feasibility and Clinical Implementation of Daily MR-Guided Adaptive Head and Neck Cancer Radiation Therapy on a 1.5T MR-Linac System: Prospective R-IDEAL 2a/2b Systematic Clinical Evaluation of Technical Innovation.	McDonald BA; Vedam S; Yang J; Wang J; Castillo P; Lee B; Sobremonte A; Ahmed S; Ding Y; Mohamed ASR; Balter P; Hughes N; Thorwarth D; Nachbar M; Philippens MEP; Terhaard CHJ; Zips D; Böke S; Awan MJ; Christodouleas J; Fuller CD	10.1016/j.ijrobp.2020.12.015	https://pubmed.ncbi.nlm.nih.gov/33340604
1.5T Magnetic Resonance-Guided Stereotactic Body Radiotherapy for Localized Prostate Cancer: Preliminary Clinical Results of Clinician- and Patient-Reported Outcomes.	Poon DMC; Yuan J; Wong OL; Yang B; Chiu ST; Cheung KY; Chiu G; Yu SK	10.3390/cancers13194866	
Online adaptive MR-guided stereotactic radiotherapy for unresectable malignancies in the upper abdomen using a 1.5T MR-linac.	Daamen LA; de Mol van Otterloo SR; van Goor IWJM; Eijkelenkamp H; Erickson BA; Hall WA; Heerkens HD; Meijer GJ; Molenaar IQ; van Santvoort HC; Verkooijen HM; Intven MPW	10.1080/0284186X.2021.2012593	https://pubmed.ncbi.nlm.nih.gov/34879792
Patterns of Care, Tolerability, and Safety of the First Cohort of Patients Treated on a Novel High-Field MR-Linac Within the MOMENTUM Study: Initial Results From a Prospective Multi-Institutional Registry.	Christodouleas JP; Blezer ELA; Akhiat H; Brown K; Choudhury A; Eggert D; Erickson BA; Daamen LA; Faivre-Finn C; Fuller CD; Goldwein J; Hafeez S; Hall E; Harrington KJ; van der Heide UA; Huddart RA; Intven MPW; Kirby AM; Lalondrelle S; McCann C; Minsky BD; Mook S; Nowee ME; Oelfke U; Orrling K; Philippens MEP; Sahgal A; Schultz CJ; Tersteeg RJHA; Tijssen RHN; Tree AC; van Triest B; Tseng CL; Hall WA; Verkooijen HM; de_Mol_van_Otterloo SR	10.1016/j.ijrobp.2021.07.003	https://pubmed.ncbi.nlm.nih.gov/34265394
First multicentre experience of SABR for lymph node and liver oligometastatic disease on the unity MR-Linac.	Janssen TM; Aitken K; Alongi F; Barry A; Bernchou U; Boeke S; Hall WA; Hosni A; Kroon PS; Nachbar M; Saeed H; Jürgenliemk-Schulz IM; Schytte T; Verkooijen HM; Nowee ME	10.1016/j.tipsro.2022.04.005	

Restricted Information and Basic Personal Data

Title	Authors	DOI	PMID URL
Initial clinical applications treating pediatric and adolescent patients using MR-guided radiotherapy.	Kozak MM; Crompton D; Gross BA; Harshman L; Dickens D; Snyder J; Shepard A; St-Aubin J; Dunkerley D; Hyer D; Buatti JM	10.3389/fonc.2022.962926	
Initial clinical experience of Stereotactic Body Radiation Therapy (SBRT) for liver metastases, primary liver malignancy, and pancreatic cancer with 4D-MRI based online adaptation and real-time MRI monitoring using a 1.5 Tesla MR-Linac.	Hall WA; Straza MW; Chen X; Mickevicius N; Erickson B; Schultz C; Awan M; Ahunbay E; Li XA; Paulson ES	10.1371/journal.pone.0236570	https://pubmed.ncbi.nlm.nih.gov/32764748
Clinical application of a sub-fractionation workflow for intrafraction re-planning during prostate radiotherapy treatment on a 1.5 Tesla MR-Linac: A practical method to mitigate intrafraction motion.	Willigenburg T; Zachiu C; Bol GH; de Groot-van Beugel EN; Lagendijk JJW; van der Voort van Zyp JRN; Raaymakers BW; de Boer JCJ	10.1016/j.radonc.2022.09.004	https://pubmed.ncbi.nlm.nih.gov/36113777
Marker-less online MR-guided stereotactic body radiotherapy of liver metastases at a 1.5 T MR-Linac - Feasibility, workflow data and patient acceptance.	Gani C; Boeke S; McNair H; Ehlers J; Nachbar M; Monnich D; Stolte A; Boldt J; Marks C; Winter J; Kunzel LA; Gatidis S; Bitzer M; Thorwarth D; Zips D	10.1016/j.ctro.2020.11.014	https://pubmed.ncbi.nlm.nih.gov/33319073
Magnetic Resonance-Guided Adaptive Radiation therapy for Prostate Cancer: The First Results from the MOMENTUM study-An International Registry for the Evidence-Based Introduction of Magnetic Resonance-Guided Adaptive Radiation Therapy.	Teunissen FR; Willigenburg T; Tree AC; Hall WA; Choi SL; Choudhury A; Christodouleas JP; de Boer JCJ; Groot-van Breugel EN; Kerkmeijer LGW; Pos FJ; Schytte T; Vesprini D; Verkooijen HM; Voort van Zyp JRNV	10.1016/j.prro.2022.09.007	https://pubmed.ncbi.nlm.nih.gov/36462619
Assessment of delivered dose in prostate cancer patients treated with ultra-hypofractionated radiotherapy on 1.5-Tesla MR-Linac.	Gao LR; Tian Y; Wang MS; Xia WL; Qin SR; Song YW; Wang SL; Tang Y; Fang H; Tang Y; Qi SN; Yan LL; Liu YP; Jing H; Chen B; Xing NZ; Li YX; Lu NN	10.3389/fonc.2023.1039901	https://pubmed.ncbi.nlm.nih.gov/36741014
Feasibility and safety of 1.5 T MR-guided and daily adapted abdominal-pelvic SBRT for elderly cancer patients: geriatric assessment tools and preliminary patient-reported outcomes.	Mazzola R; Figlia V; Rigo M; Cuccia F; Ricchetti F; Giaj-Levra N; Nicosia L; Vitale C; Sicignano G; De Simone A; Naccarato S; Ruggieri R; Alongi F	10.1007/s00432-020-03230-w	https://pubmed.ncbi.nlm.nih.gov/32372146
Early experience with MR-guided adaptive radiotherapy using a 1.5 T MR-Linac: First 6 months of operation using adapt to shape workflow.	de Leon J; Crawford D; Moutrie Z; Alvares S; Hogan L; Pagulayan C; Jelen U; Loo C; Aylward JD; Condon K; Dunkerley N; Heinke MY; Sampaio S; Simon K; Twentyman T; Jameson MG	10.1111/1754-9485.13336	https://pubmed.ncbi.nlm.nih.gov/34643065
Clinical Implementational and Site-Specific Workflows for a 1.5T MR-Linac.	Dunkerley DAP; Hyer DE; Snyder JE; St-Aubin JJ; Anderson CM; Caster JM; Smith MC; Buatti JM; Yaddanapudi S	10.3390/jcm11061662	https://pubmed.ncbi.nlm.nih.gov/35329988
Feasibility of ablative stereotactic body radiation therapy of pancreas cancer patients on a 1.5 Tesla magnetic	Tyagi N; Liang J; Burleson S; Subashi E; Godoy Scripes P; Tringale	10.1016/j.phro.2021.07.006	https://pubmed.ncbi.nlm.nih.gov/34307919

Restricted Information and Basic Personal Data

Title	Authors	DOI	PMID URL
resonance-linac system using abdominal compression.	KR; Romesser PB; Reyngold M; Crane CH		
Online adaptive MR-guided radiotherapy for rectal cancer; feasibility of the workflow on a 1.5T MR-linac: clinical implementation and initial experience.	Intven MPW; de Mol van Otterloo SR; Mook S; Doornaert PAH; de Groot-van Breugel EN; Sikkes GG; Willemsen-Bosman ME; van Zijp HM; Tijssen RHN	10.1016/j.radonc.2020.09.024	https://pubmed.ncbi.nlm.nih.gov/32976875
Clinical implementation and feasibility of long-course fractionated MR-guided chemoradiotherapy for patients with esophageal cancer: An R-IDEAL stage 1b/2a evaluation of technical innovation.	Boekhoff MR; Bouwmans R; Doornaert PAH; Intven MPW; Lagendijk JJW; van Lier ALHMW; Rasing MJA; van de Ven S; Meijer GJ; Mook S	10.1016/j.ctro.2022.03.008	https://pubmed.ncbi.nlm.nih.gov/35372703
Stereotactic body radiotherapy for oligometastatic castration sensitive prostate cancer using 1.5 T MRI-Linac: preliminary data on feasibility and acute patient-reported outcomes.	Mazzola R; Cuccia F; Figlia V; Rigo M; Nicosia L; Giaj-Levra N; Ricchetti F; Vitale C; Mantoan B; Di Paola G; De Simone A; Gurrera D; Sicignano G; Naccarato S; Ruggieri R; Alongi F	10.1007/s11547-021-01352-w	
1.5T MR-Guided Daily-Adaptive SBRT for Prostate Cancer: Preliminary Report of Toxicity and Quality of Life of the First 100 Patients.	Alongi F; Rigo M; Figlia V; Nicosia L; Mazzola R; Giaj Levra N; Ricchetti F; Trapani G; Attinà G; Vitale C; Pastorello E; De Simone A; Gurrera D; Naccarato S; Sicignano G; Ruggieri R; Cuccia F	10.3390/jpm12121982	https://pubmed.ncbi.nlm.nih.gov/36556203
Stereotactic ablative radiation for pancreatic cancer on a 1.5 Telsa magnetic resonance-linac system.	Tringale KR; Tyagi N; Marsha Reyngold; Romesser PB; Wu A; O'Reilly EM; Varghese AM; Godoy Scripes P; Khalil DN; Park W; Yu K; Crane CH	10.1016/j.phro.2022.10.003	https://pubmed.ncbi.nlm.nih.gov/36386447
1.5 T MR-Guided Daily Adapted SBRT on Lymph Node Oligometastases from Prostate Cancer.	Nicosia L; Trapani G; Rigo M; Giaj-Levra N; Mazzola R; Pastorello E; Ricchetti F; Cuccia F; Figlia V; Fiorini M; Alongi F	10.3390/jcm11226658	https://pubmed.ncbi.nlm.nih.gov/36431135
Analysis of online plan adaptation for 1.5T magnetic resonance-guided stereotactic body radiotherapy (MRgSBRT) of prostate cancer.	Poon DMC; Yang B; Geng H; Wong OL; Chiu ST; Cheung KY; Yu SK; Chiu G; Yuan J	10.1007/s00432-022-03950-1	
Technical feasibility and clinical evaluation of 4D-MRI guided liver SBRT on the MR-linac.	van de Lindt A; Nowee B; Janssen T; Schneider C; Remeijer P; van Pelt VWJ; Betgen A; Jansen EPM; Sonke JJ	10.1016/j.radonc.2022.01.009	https://pubmed.ncbi.nlm.nih.gov/35033603
First Experience and Prospective Evaluation on Feasibility and Acute Toxicity of Online Adaptive Radiotherapy of the Prostate Bed as Salvage Treatment in Patients with Biochemically Recurrent Prostate Cancer on a 1.5T MR-Linac.	Wegener D; Thome A; Paulsen F; Gani C; Boldt J; Butzer S; Thorwarth D; Moennich D; Nachbar M; Müller AC; Zips D; Boeke S	10.3390/jcm11164651	https://pubmed.ncbi.nlm.nih.gov/36012885
A Prospective Study of Stereotactic Body Radiotherapy (SBRT) with Concomitant Whole-Pelvic Radiotherapy (WPRT) for High-Risk Localized Prostate Cancer Patients Using 1.5 Tesla Magnetic Resonance	Poon DMC; Yuan J; Yang B; Wong OL; Chiu ST; Chiu G; Cheung KY; Yu SK; Yung RWH	10.3390/cancers14143484	

Restricted Information and Basic Personal Data

Title	Authors	DOI	PMID URL
Guidance: The Preliminary Clinical Outcome.			
Individual lymph nodes: "See it and Zap it".	Winkel D; Werensteijn-Honingh AM; Kroon PS; Eppinga WSC; Bol GH; Intven MPW; de Boer HCJ; Snoeren LMW; Hes J; Raaymakers BW; Jurgenliemk-Schulz IM	10.1016/j.ctro.2019.03.004	https://pubmed.ncbi.nlm.nih.gov/31341975
Clinical Implementation and Initial Experience With a 1.5 Tesla MR-Linac for MR-Guided Radiation Therapy for Gynecologic Cancer: An R-IDEAL Stage 1 and 2a First in Humans Feasibility Study of New Technology Implementation.	Lakomy DS; Yang J; Vedam S; Wang J; Lee B; Sobremonte A; Castillo P; Hughes N; Mohammedsaid M; Jhingran A; Klopp AH; Choi S; Fuller CD; Lin LL	10.1016/j.prro.2022.03.002	https://pubmed.ncbi.nlm.nih.gov/35278717
Development of patient-reported outcomes item set to evaluate acute treatment toxicity to pelvic online magnetic resonance-guided radiotherapy.	Moller PK; Pappot H; Bernchou U; Schytte T; Dieperink KB	10.1186/s41687-021-00326-w	
1.5 T MR-guided and daily adapted SBRT for prostate cancer: feasibility, preliminary clinical tolerability, quality of life and patient-reported outcomes during treatment.	Alongi F; Rigo M; Figlia V; Cuccia F; Giaj-Levra N; Nicosia L; Ricchetti F; Sicignano G; De Simone A; Naccarato S; Ruggieri R; Mazzola R	10.1186/s13014-020-01510-w	https://pubmed.ncbi.nlm.nih.gov/32248826
Local control and patient reported outcomes after online MR guided stereotactic body radiotherapy of liver metastases.	Uder L; Nachbar M; Butzer S; Boldt J; Baumeister S; Bitzer M; Königsrainer A; Seufferlein T; Hoffmann R; Gatidis S; Nikolaou K; Zips D; Thorwarth D; Gani C; Boeke S	10.3389/fonc.2022.1095633	https://pubmed.ncbi.nlm.nih.gov/36727060
Linac-based versus MR-guided SBRT for localized prostate cancer: a comparative evaluation of acute tolerability.	Nicosia L; Mazzola R; Rigo M; Giaj-Levra N; Pastorello E; Ricchetti F; Vitale C; Figlia V; Cuccia F; Ruggieri R; Alongi F	10.1007/s11547-023-01624-7	https://pubmed.ncbi.nlm.nih.gov/37055672
Effectiveness of bladder filling control during online MR-guided adaptive radiotherapy for rectal cancer.	Feng X; Tang B; Yao X; Liu M; Liao X; Yuan K; Peng Q; Orlandini LC	10.1186/s13014-023-02315-3	https://pubmed.ncbi.nlm.nih.gov/37592338
Interim toxicity analysis from the randomised HERMES trial of 2- and 5-fraction MRI-guided adaptive prostate radiotherapy.	Westley DRL; Biscombe K; Dunlop A; Mitchell A; Oelfke U; Nil S; Murray J; Pathmanathan A; Hafeez S; Parker C; Ratnakumaran R; Alexander S; Herbert T; Hall E; Tree AC	10.1016/j.ijrobp.2023.09.032	https://pubmed.ncbi.nlm.nih.gov/37776979
Clinical outcomes after online adaptive MR-guided stereotactic body radiotherapy for pancreatic tumors on a 1.5 T MR-linac	Eijkelenkamp Hidde; Grimbergen Guus; Daamen Lois; Heerkens Hanne; van de Ven Saskia; Mook Stella; Meijer Gert; Molenaar Izaak; van Santvoort Hjalmar; Paulson Eric; Erickson Beth; Verkooijen Helena; Hall William; Intven Martijn	10.3389/fonc.2023.1040673	
Pathological and surgical outcomes of pancreatic adenocarcinoma (PA) after pre-operative ablative stereotactic magnetic resonance image-guided adaptive radiotherapy (A-SMART)	Bryant JM; Palm Russell F; Liveringhouse Casey; Boyer Emanuel; Hodul Pam; Malafa Mokenge; Denbo Jason; Kim Dae;	10.1016/j.adro.2022.101045	

Restricted Information and Basic Personal Data

Title	Authors	DOI	PMID URL
	Carballido Estrella; Fleming Jason B; Hoffe Sarah; Frakes Jessica		
Magnetic Resonance-guided Stereotactic Radiotherapy for Localized Prostate Cancer: Final Results on Patient-reported Outcomes of a Prospective Phase 2 Study.	Tetar SU; Bruynzeel AME; Oei SS; Senan S; Fraikin T; Slotman BJ; Moorselaar RJAV; Lagerwaard FJ	10.1016/j.euo.2020.05.007	
Patient-reported Outcome Measurements on the Tolerance of Magnetic Resonance Imaging-guided Radiation Therapy.	Tetar S; Bruynzeel A; Bakker R; Jeulink M; Slotman BJ; Oei S; Haasbeek C; De Jong K; Senan S; Lagerwaard F	10.7759/cureus.2236	
Stereotactic body radiotherapy of lymph node metastases under MR-guidance: First clinical results and patient-reported outcomes.	Weykamp F; Herder-Wagner C; Regnery S; Hoegen P; Renkamp CK; Liermann J; Rippke C; Koerber SA; Konig L; Buchele C; Kluter S; Debus J; Horner-Rieber J	10.1007/s00066-021-01834-w	
The Role of Daily Adaptive Stereotactic MR-Guided Radiotherapy for Renal Cell Cancer.	Tetar SU; Bohoudi O; Senan S; Palacios MA; Oei SS; Wel AMV; Slotman BJ; Moorselaar RJAV; Lagerwaard FJ; Bruynzeel AME	10.3390/cancers12102763	
Stereotactic MR-Guided Radiotherapy for Adrenal Gland Metastases: First Clinical Results.	Michalet M; Bettaïeb O; Khalfi S; Ghorbel A; Valdenaire S; Debuire P; Aillères N; Draghici R; De Méric De Bellefon M; Charissoux M; Boisselier P; Demontoy S; Marguerit A; Cabaillé M; Cantaloube M; Keskes A; Bouhafa T; Farcy-Jacquet MP; Fenoglietto P; Azria D; Riou O	10.3390/jcm12010291	https://pubmed.ncbi.nlm.nih.gov/36615093
Operating procedures, risk management and challenges during implementation of adaptive and non-adaptive MR-guided radiotherapy: 1-year single-center experience.	Garcia Schuler HI; Pavic M; Mayinger M; Weitkamp N; Chamberlain M; Reiner C; Linsenmeier C; Balermpas P; Krayenbuhl J; Guckenberger M; Baumgartl M; Wilke L; Tanadini-Lang S; Andratschke N	10.1186/s13014-021-01945-9	https://pubmed.ncbi.nlm.nih.gov/34775998
A pilot study of same-day MRI-only simulation and treatment with MR-guided adaptive palliative radiotherapy (MAP-RT)	Schiff Joshua P.; Maraghechi Borna; Chin Re-I.; Price Alex; Laugeman Eric; Rudra Souman; Hatscher Casey; Spraker Matthew B.; Badiyan Shahed N.; Henke Lauren E.; Green Olga; Kim Hyun	10.1016/j.ctro.2022.100561	
Implementation of Stereotactic MRI-Guided Adaptive Radiotherapy (SMART) for Hepatobiliary and Pancreatic Cancers in the United Kingdom - Fifty in Five.	Gaya A; Camilleri P; Nash A; Hughes D; Good J	10.7759/cureus.15075	https://pubmed.ncbi.nlm.nih.gov/34150409
Single-Institution Phase 1/2 Prospective Clinical Trial of Single-Fraction, High-Gradient Adjuvant Partial-Breast Irradiation for Hormone Sensitive Stage 0-I Breast Cancer.	Kennedy WR; Thomas MA; Stanley JA; Luo J; Ochoa LL; Clifton KK; Cyr AE; Margenthaler JA; DeWees TA; Price A; Kashani R; Green O; Zoberi I	10.1016/j.ijrobp.2020.02.021	
SMART ablation of lymphatic oligometastases in the pelvis and abdomen: Clinical and dosimetry outcomes.	Regnery S; Buchele C; Piskorski L; Weykamp F; Held T; Eichkorn T; Rippke C; Katharina Renkamp C; Kluter S; Ristau J; Konig L; Koerber	10.1016/j.radonc.2022.01.038	

Restricted Information and Basic Personal Data

Title	Authors	DOI	PMID URL
	SA; Adeberg S; Debus J; Horner-Rieber J		
Stereotactic MR-Guided Radiotherapy for Liver Metastases: First Results of the Montpellier Prospective Registry Study.	Bordeau K; Michalet M; Keskes A; Valdenaire S; Debuire P; Cantaloube M; Cabaillé M; Jacot W; Draghici R; Demontoy S; Quantin X; Ychou M; Assenat E; Mazard T; Gauthier L; Dupuy M; Guiu B; Bourgier C; Aillères N; Fenoglietto P; Azria D; Riou O	10.3390/jcm12031183	
Implementing Stereotactic Accelerated Partial Breast Irradiation using Magnetic Resonance Guided Radiation Therapy.	Price AT; Kennedy WR; Henke LE; Brown SR; Green OL; Thomas MA; Zoberi I	10.1016/j.radonc.2021.09.023	
Same-day consultation, simulation and lung SABR delivery on an MR-linac	Palacios M.A.; Verheijen S.; Schneiders F.S.C.; Bohoudi O.; Slotman B.J.; Lagerwaard F.J.; Senan S.	10.1016/j.phro.2022.09.010	
Application of real-time MRI-guided linear accelerator in stereotactic ablative body radiotherapy for non-small cell lung cancer: one step forward to precise targeting.	Kang HJ; Kwak YK; Kim M; Lee SJ	10.1007/s00432-022-04264-y	
Magnetic Resonance-Guided Reirradiation for Local Recurrence Within the Prostate or in the Prostate Bed: Preliminary Results of a Prospective Registry Study.	Michalet M; Riou O; Valdenaire S; Debuire P; Ailleres N; Draghici R; Charissoux M; Moscardo CL; Farcy-Jacquet MP; Fenoglietto P; Azria D	10.1016/j.adro.2021.100748	
Quality-of-Life Outcomes and Toxicity Profile Among Patients With Localized Prostate Cancer After Radical Prostatectomy Treated With Stereotactic Body Radiation: The SCIMITAR Multicenter Phase 2 Trial.	Ma TM; Ballas LK; Wilhalme H; Sachdeva A; Chong N; Sharma S; Yang T; Basehart V; Reiter RE; Saigal C; Chamie K; Litwin MS; Rettig MB; Nickols NG; Yoon SM; Smith L; Gao Y; Steinberg ML; Cao M; Kishan AU	10.1016/j.ijrobp.2022.08.041	
Dose accumulation for personalized stereotactic MR-guided adaptive radiation therapy in prostate cancer.	Bohoudi O; Bruynzeel AME; Tetar S; Slotman BJ; Palacios MA; Lagerwaard FJ	10.1016/j.radonc.2021.01.022	
Renal atrophy following gated delivery of stereotactic ablative radiotherapy to adrenal metastases.	van Sornsen de Koste JR; van Vliet CC; Schneiders FL; Bruynzeel AME; Slotman BJ; Palacios MA; Senan S	10.1016/j.phro.2021.09.001	
Stereotactic magnetic resonance-guided online adaptive radiotherapy of adrenal metastases combines high ablative doses with optimized sparing of organs at risk.	Hoegen Philipp; Katsigiannopulos Efthimios; Buchele Carolin; Regnery Sebastian; Weykamp Fabian; Sandrini Elisabetta; Ristau Jonas; Liermann Jakob; Meixner Eva; Forster Tobias; Renkamp C. Katharina; Schlüter Fabian; Rippke Carolin; Debus Jürgen; Klüter Sebastian; Hörner-Rieber Juliane	10.1016/j.ctro.2022.100567	https://pubmed.ncbi.nlm.nih.gov/36935853
Ablative radiotherapy for liver tumors using stereotactic MRI-guidance: A prospective phase I trial.	van Dams R; Wu TC; Kishan AU; Raldow AC; Chu FI; Hernandez J; Cao M; Lamb JM; Mikaeilian A; Low DA; Steinberg ML; Lee P	10.1016/j.radonc.2021.06.005	

Restricted Information and Basic Personal Data

Title	Authors	DOI	PMID URL
Magnetic Resonance Imaging-Guided vs Computed Tomography-Guided Stereotactic Body Radiotherapy for Prostate Cancer: The MIRAGE Randomized Clinical Trial.	Kishan AU; Ma TM; Lamb JM; Casado M; Wilhalme H; Low DA; Sheng K; Sharma S; Nickols NG; Pham J; Yang Y; Gao Y; Neylon J; Basehart V; Cao M; Steinberg ML	10.1001/jamaoncol.2022.6558	
First 500 Fractions Delivered with a Magnetic Resonance-guided Radiotherapy System: Initial Experience.	Sahin B; Zoto Mustafayev T; Gungor G; Aydin G; Yapici B; Atalar B; Ozyar E	10.7759/cureus.6457	https://pubmed.ncbi.nlm.nih.gov/32025388
[Start of activity with the MRIdian(R) system: The first 200 patients treated at the Institut Paoli-Calmettes].	Tyran M; Fau P; Mailleux H; Eustache P; Benkreira M; Salem N; Favrel V; Gonzague L; Moureau-Zabotto L; Varela L; Annede P; Tallet A	10.1016/j.bulcan.2021.05.013	
MR-guided radiation therapy with concurrent gemcitabine / nab-paclitaxel chemotherapy in inoperable pancreatic cancer: a TITE-CRM phase I trial.	Kim H; Olsen JR; Green OL; Chin RI; Hawkins WG; Fields RC; Hammill C; Doyle MB; Chapman W; Suresh R; Tan B; Pedersen K; Jansen B; DeWees TA; Lu E; Henke LE; Badiyan S; Parikh PJ; Roach MC; Wang-Gillam A; Lim KH	10.1016/j.ijrobp.2022.07.015	
Phase I trial of stereotactic MR-guided online adaptive radiation therapy (SMART) for the treatment of oligometastatic or unresectable primary malignancies of the abdomen.	Henke L; Kashani R; Robinson C; Curcuru A; DeWees T; Bradley J; Green O; Michalski J; Mutic S; Parikh P; Olsen J	10.1016/j.radonc.2017.11.032	
Phase I Trial of Stereotactic MRI-Guided Online Adaptive Radiation Therapy (SMART) for the Treatment of Oligometastatic Ovarian Cancer.	Henke LE; Stanley JA; Robinson C; Srivastava A; Contreras JA; Curcuru A; Green OL; Massad LS; Kuroki L; Fuh K; Hagemann A; Mutch D; McCourt C; Thaker P; Powell M; Markovina S; Grigsby PW; Schwarz JK; Chundury A	10.1016/j.ijrobp.2021.08.033	
Magnetic Resonance-Guided Stereotactic Body Radiotherapy of Liver Tumors: Initial Clinical Experience and Patient-Reported Outcomes.	Weykamp F; Hoegen P; Kluter S; Spindeldreier CK; Konig L; Seidensaal K; Regnery S; Liermann J; Rippke C; Koerber SA; Buchele C; Debus J; Horner-Rieber J	10.3389/fonc.2021.610637	
Stereotactic magnetic resonance-guided online adaptive radiotherapy of adrenal metastases combines high ablative doses with optimized sparing of organs at risk.	Hoegen P; Katsigiannopulos E; Buchele C; Regnery S; Weykamp F; Sandrini E; Ristau J; Liermann J; Meixner E; Forster T; Renkamp CK; Schlüter F; Rippke C; Debus J; Klüter S; Hörner-Rieber J	10.1016/j.ctro.2022.100567	https://pubmed.ncbi.nlm.nih.gov/36935853
Is MRI-Linac helpful in SABR treatments for liver cancer?	Tallet A; Boher JM; Tyran M; Mailleux H; Piana G; Benkreira M; Fau P; Salem N; Gonzague L; Petit C; Darréon J	10.3389/fonc.2023.1130490	https://pubmed.ncbi.nlm.nih.gov/37007109
Magnetic resonance-guided stereotactic body radiation therapy (MRgSBRT) for oligometastatic patients: a single-center experience.	Chiloiro G; Boldrini L; Romano A; Placidi L; Tran HE; Nardini M; Massaccesi M; Cellini F; Indovina L; Gambacorta MA	10.1007/s11547-023-01627-4	https://pubmed.ncbi.nlm.nih.gov/37079221
Clinical outcomes of patients with unresectable primary liver cancer treated with MR-guided stereotactic	Chin RI; Schiff JP; Bommireddy A; Kang KH; Andruska N; Price AT; Green OL; Huang Y; Korenblat K;	10.1016/j.ctro.2023.100627	https://pubmed.ncbi.nlm.nih.gov/37441543

Restricted Information and Basic Personal Data

Title	Authors	DOI	PMID URL
body radiation Therapy: A Six-Year experience.	Parikh PJ; Olsen J; Samson PP; Henke LE; Kim H; Badiyan SN		
A Multi-Institutional Phase 2 Trial of Ablative 5-Fraction Stereotactic Magnetic Resonance-Guided On-Table Adaptive Radiation Therapy for Borderline Resectable and Locally Advanced Pancreatic Cancer.	Parikh PJ; Lee P; Low DA; Kim J; Mittauer KE; Bassetti MF; Glide-Hurst CK; Raldow AC; Yang Y; Portelance L; Padgett KR; Zaki B; Zhang R; Kim H; Henke LE; Price AT; Mancias JD; Williams CL; Ng J; Pennell R; Pfeffer MR; Levin D; Mueller AC; Mooney KE; Kelly P; Shah AP; Boldrini L; Placidi L; Fuss M; Chuong MD	10.1016/j.ijrobp.2023.05.023	https://pubmed.ncbi.nlm.nih.gov/37210048
Magnetic resonance guided SBRT reirradiation in locally recurrent prostate cancer: a multicentric retrospective analysis.	Boldrini L; Romano A; Chiloiro G; Corradini S; De Luca V; Verusio V; D'Aviero A; Castelluccia A; Alitto AR; Catucci F; Grimaldi G; Trapp C; Hörner-Rieber J; Marchesano D; Frascino V; Mattiucci GC; Valentini V; Gentile P; Gambacorta MA	10.1186/s13014-023-02271-y	https://pubmed.ncbi.nlm.nih.gov/37218005
Tumor volume changes during stereotactic ablative radiotherapy for adrenal gland metastases under MRI guidance.	Giraud N; Schneiders FL; van Sornsen de Koste JR; Palacios MA; Senan S	10.1016/j.radonc.2023.109749	https://pubmed.ncbi.nlm.nih.gov/37330058
Safety and Efficacy of Neoadjuvant Stereotactic Ablative Radiotherapy (SABR) in Pancreatic Cancer: Impact of Magnetic Resonance Imaging (MRI)-Guided Respiratory-Gated Adaptive Radiotherapy	Song Jun; Kim Yong-Tae; Ryu Ji; Lee Sang; Paik Woo; Cho In; Kim Hongbeom; Kwon Wooil; Jang Jin-Young; Chie Eui; Kang Hyun-Cheol	10.1016/j.adro.2023.101312	
Acute toxicity comparison of magnetic resonance-guided adaptive versus fiducial or computed tomography-guided non-adaptive prostate stereotactic body radiotherapy: A systematic review and meta-analysis.	Leeman JE; Shin KY; Chen YH; Mak RH; Nguyen PL; D'Amico AV; Martin NE	10.1002/cncr.34836	https://pubmed.ncbi.nlm.nih.gov/37485697
Stereotactic Magnetic Resonance-guided Adaptive Radiation Therapy for Localized Kidney Cancer: Early Outcomes from a Prospective Phase 1 Trial and Supplemental Cohort.	Yim K; Hsu SH; Nolazco J; Cagney D; Mak RH; D'Andrea V; Singer L; Williams C; Huynh E; Han Z; Martin N; Nguyen P; Kibel AS; Choueiri TK; Chang SL; Leeman JE	10.1016/j.euo.2023.07.002	https://pubmed.ncbi.nlm.nih.gov/37487813
Stereotactic MR-guided adaptive radiotherapy (SMART) for primary rectal cancer: evaluation of early toxicity and pathological response	Castelluccia, Alessandra	10.5603/RPOR.a2023.0051	https://pubmed.ncbi.nlm.nih.gov/37795221
Local control and toxicity after magnetic resonance imaging (MR)-guided single fraction lung stereotactic ablative radiotherapy.	Tekatli H; Palacios MA; Schneiders FL; Haasbeek CJ; Haasbeek CJ; Slotman BJ; Lagerwaard FJ; Senan S	10.1016/j.radonc.2023.109823	https://pubmed.ncbi.nlm.nih.gov/37516364
Clinical outcomes of MR-guided adrenal stereotactic ablative	Schneiders Famke; van Vliet Claire; Giraud Nicolas; Bruynzeel Anna;	10.1016/j.ctro.2023.100680	

Restricted Information and Basic Personal Data

Title	Authors	DOI	PMID URL
radiotherapy with preferential sparing of organs at risk	Slotman Ben; Palacios Miguel; Senan Suresh		
Online Adaptive Magnetic Resonance-guided Radiation Therapy for Gynaecological Cancers: Preliminary Results of Feasibility and Outcome	Ugurluer G.; Zoto Mustafayev T.; Gungor G.; Abacioglu U.; Atalar B.; Ozyar E.	10.1016/j.clon.2023.11.036	

Supplementary Table S4: CBCTgART Clinical Publications

Title	Authors	DOI	PMID URL
Online adaptive radiotherapy of urinary bladder cancer with full re-optimization to the anatomy of the day: initial experience and dosimetric benefits.	Astrom LM,Behrens CP,Calmels L,Sjostrom D,Geertsen P,Mouritsen LS,Serup-Hansen E,Lindberg H,Sibolt P	10.1016/j.radonc.2022.03.014	https://pubmed.ncbi.nlm.nih.gov/35358605/
Online adaptive radiotherapy of anal cancer: normal tissue sparing, target propagation methods, and first clinical experience.	Ã…strÃ¶m LM,Behrens CP,Smedegaard Storm K,Sibolt P,Serup-Hansen E	10.1016/j.radonc.2022.09.015	https://pubmed.ncbi.nlm.nih.gov/36174846/
Feasibility of Conebeam CT-based online adaptive radiotherapy for neoadjuvant treatment of rectal cancer.	de Jong R,Visser J,van Wieringen N,Wiersma J,Geijsen D,Bel A	10.1186/s13014-021-01866-7	https://pubmed.ncbi.nlm.nih.gov/34301300/
Same-day adaptive palliative radiotherapy without prior CT simulation: Early outcomes in the FAST-METS study.	Joshua Nelissen K,Versteijne E,Senan S,Rijksen B,Admiraal M,Visser J,Barink S,Lisa de la Fuente A,Hoffmans D,Slotman BJ,F A R Verbakel W	10.1016/j.radonc.2023.109538	https://pubmed.ncbi.nlm.nih.gov/36806603/
Cone-beam computed tomography-guided online adaptive radiotherapy is feasible for prostate cancer patients.	Zwart LGM,Ong F,Ten Asbroek LA,van Dieren EB,Koch SA,Bhawanie A,de Wit E,Dasselaar JJ	10.1016/j.phro.2022.04.009	https://pubmed.ncbi.nlm.nih.gov/35602545/
Varian ethos online adaptive radiotherapy for prostate cancer: Early results of contouring accuracy, treatment plan quality, and treatment time.	Byrne M,Archibald-Heeren B,Hu Y,Teh A,Beserminji R,Cai E,Liu G,Yates A,Rijken J,Collett N,Aland T	10.1002/acm2.13479	https://pubmed.ncbi.nlm.nih.gov/34846098/
Clinical Experience in Prostate Ultrahypofractionated Radiation Therapy With an Online Adaptive Method	Calvo-Ortega JF; Moragues-Femenía S; Laosa-Bello C; Torices-Caballero J; Hermida-López M; Casals-Farran J	10.1016/j.prro.2021.10.001	
Long-term Follow-up Results of CT-guided Daily Adaptive Radiation Therapy for Localized Prostate Cancer.	Hama Y; Kaji T	10.21873/anticanres.12942	
Reduction of PTV margins for elective pelvic lymph nodes in online adaptive radiotherapy of prostate cancer patients.	Brennsæter JA; Dahle TJ; Moi JN; Svanberg IF; Haaland GS; Pilskog S	10.1080/0284186X.2023.2252584	https://pubmed.ncbi.nlm.nih.gov/37682727
Online adaptive radiotherapy for bladder cancer using a simultaneous integrated boost and fiducial markers.	Azzarouali S; Goudschaal K; Visser J; Hulshof M; Admiraal M; van Wieringen N; Nieuwenhuijzen J; Wiersma J; Daniëls L; den Boer D; Bel A	10.1186/s13014-023-02348-8	https://pubmed.ncbi.nlm.nih.gov/37803392

Restricted Information and Basic Personal Data

Supplementary Table S5: PBT Randomized Control Trial Publications

Title	Authors	DOI	PMID URL
A prospective phase II randomized trial of proton radiotherapy vs intensity-modulated radiotherapy for patients with newly diagnosed glioblastoma.	Brown PD,Chung C,Liu DD,McAvoy S,Grosshans D,Al Feghali K,Mahajan A,Li J,McGovern SL,McAleer MF,Ghia AJ,Sulman EP,Penas-Prado M,de Groot JF,Heimberger AB,Wang J,Armstrong TS,Gilbert MR,Guha-Thakurta N,Wefel JS	10.1093/neuonc/noab040	https://pubmed.ncbi.nlm.nih.gov/33647972/
Randomized trial comparing conventional-dose with high-dose conformal radiation therapy in early-stage adenocarcinoma of the prostate: long-term results from proton radiation oncology group/american college of radiology 95-09.	Zietman AL,Bae K,Slater JD,Shipley WU,Efstathiou JA,Coen JJ,Bush DA,Lunt M,Spiegel DY,Skowronski R,Jabola BR,Rossi CJ	10.1200/JCO.2009.25.8475	https://pubmed.ncbi.nlm.nih.gov/20124169/
Proton beam radiotherapy vs. radiofrequency ablation for recurrent hepatocellular carcinoma: A randomized phase III trial.	Kim TH,Koh YH,Kim BH,Kim MJ,Lee JH,Park B,Park JW	10.1016/j.jhep.2020.09.026	https://pubmed.ncbi.nlm.nih.gov/33031846/
Randomized Phase IIB Trial of Proton Beam Therapy Versus Intensity-Modulated Radiation Therapy for Locally Advanced Esophageal Cancer.	Lin SH,Hobbs BP,Verma V,Tidwell RS,Smith GL,Lei X,Corsini EM,Mok I,Wei X,Yao L,Wang X,Komaki RU,Chang JY,Chun SG,Jeter MD,Swisher SG,Ajani JA,Blum-Murphy M,Vaporciyan AA,Mehran RJ,Koong AC,Gandhi SJ,Hofstetter WL,Hong TS,Delaney TF,Liao Z,Mohan R	10.1200/JCO.19.02503	https://pubmed.ncbi.nlm.nih.gov/32160096/
Bayesian Adaptive Randomization Trial of Passive Scattering Proton Therapy and Intensity-Modulated Photon Radiotherapy for Locally Advanced Non-Small-Cell Lung Cancer.	Liao Z,Lee JJ,Komaki R,Gomez DR,O'Reilly MS,Fossella FV,Blumenschein GR,Heymach JV,Vaporciyan AA,Swisher SG,Allen PK,Choi NC,DeLaney TF,Hahn SM,Cox JD,Lu CS,Mohan R	10.1200/JCO.2017.74.0720	https://pubmed.ncbi.nlm.nih.gov/29293386/
Advanced prostate cancer: the results of a randomized comparative trial of high dose irradiation boosting with conformal protons compared with conventional dose irradiation using photons alone.	Shipley WU,Verhey LJ,Munzenrider JE,Suit HD,Urie MM,McManus PL,Young RH,Shipley JW,Zietman AL,Biggs PJ	10.1016/0360-3016(95)00063-5	https://pubmed.ncbi.nlm.nih.gov/7721636/
Randomized Phase II Trial of Proton Craniospinal Irradiation Versus Photon Involved-Field Radiotherapy for Patients With Solid Tumor Leptomeningeal Metastasis.	Yang JT,Wijetunga NA,Pentsova E,Wolden S,Young RJ,Correa D,Zhang Z,Zheng J,Steckler A,Bucwinska W,Bernstein A,Betof Warner A,Yu H,Kris MG,Seidman AD,Wilcox JA,Malani R,Lin A,DeAngelis LM,Lee NY,Powell SN,Boire A	10.1200/JCO.22.01148	https://pubmed.ncbi.nlm.nih.gov/35802849/

Restricted Information and Basic Personal Data

Title	Authors	DOI	PMID URL
Work Outcomes after Intensity-Modulated Proton Therapy (IMPT) versus Intensity-Modulated Photon Therapy (IMRT) for Oropharyngeal Cancer.	Smith GL,Fu S,Ning MS,Nguyen DK,Busse PM,Foote RL,Garden AS,Gunn GB,Fuller CD,Morrison WH,Chronowski GM,Shah SJ,Mayo LL,Phan J,Reddy JP,Snider JW,Patel SH,Katz SR,Lin A,Mohammed N,Dagan R,Lee NY,Rosenthal DI,Frank SJ	10.14338/IJPT-20-00067.1	https://pubmed.ncbi.nlm.nih.gov/34285958/

Supplementary Table S6: IMRT Randomized Control Trial Publications

Title	Authors	DOI	PMID URL
Radiation Therapy Techniques and Treatment-Related Toxicity in the PORTEC-3 Trial: Comparison of 3-Dimensional Conformal Radiation Therapy Versus Intensity-Modulated Radiation Therapy.	Wortman BG,Post CCB,Powell ME,Khaw P,Fyles A,D'Amico R,Haie-Meder C,JÃ¼rgenliemk-Schulz IM,McCormack M,Do V,Katsaros D,Bessette P,Baron MH,Nout RA,Whitmarsh K,Mileshkin L,Lutgens LCHW,Kitchener HC,Brooks S,Nijman HW,Astreinidou E,Putter H,Creutzberg CL,de Boer SM	10.1016/j.ijrobp.2021.09.042	https://pubmed.ncbi.nlm.nih.gov/34610387/
Accelerated partial breast irradiation using intensity modulated radiotherapy versus whole breast irradiation: Health-related quality of life final analysis from the Florence phase 3 trial.	Meattini I,Saieva C,Miccinesi G,Desideri I,Francolini G,Scotti V,Marrazzo L,Pallotta S,Meacci F,Muntoni C,Bendinelli B,Sanchez LJ,Bernini M,Orzalesi L,Nori J,Bianchi S,Livi L	10.1016/j.ejca.2017.01.023	https://pubmed.ncbi.nlm.nih.gov/28262584/
Three-dimensional conformal radiotherapy (3D-CRT) versus intensity modulated radiation therapy (IMRT) in squamous cell carcinoma of the head and neck: a randomized controlled trial.	Gupta T,Agarwal J,Jain S,Phurailatpam R,Kannan S,Ghosh-Laskar S,Murthy V,Budrukkar A,Dinshaw K,Prabhash K,Chaturvedi P,D'Cruz A	10.1016/j.radonc.2012.07.001	https://pubmed.ncbi.nlm.nih.gov/22853852/
Accelerated Partial-Breast Irradiation Compared With Whole-Breast Irradiation for Early Breast Cancer: Long-Term Results of the Randomized Phase III APBI-IMRT-Florence Trial.	Meattini I,Marrazzo L,Saieva C,Desideri I,Scotti V,Simontacchi G,Bonomo P,Greto D,Mangoni M,Scoccianti S,Lucidi S,Paoletti L,Fambrini M,Bernini M,Sanchez L,Orzalesi L,Nori J,Bianchi S,Pallotta S,Livi L	10.1200/JCO.20.00650	https://pubmed.ncbi.nlm.nih.gov/32840419/
Comparison between Pelvic IMRT and 3D-CRT in Combination with Chemotherapy via Nrf2 Expression on the High-Risk Endometrial Cancer.	Wang N,Zhang X,Xu S,Li X,Meng Y,Dou Z	10.14715/cmb/2021.67.6.35	https://pubmed.ncbi.nlm.nih.gov/35818187/
Ten years results of the Canadian breast intensity modulated radiation therapy (IMRT) randomized controlled trial.	Pignol JP,Truong P,Rakovitch E,Sattler MG,Whelan TJ,Olivotto IA	10.1016/j.radonc.2016.08.021	https://pubmed.ncbi.nlm.nih.gov/27637858/
Prospective randomized study of intensity-modulated radiotherapy on salivary gland function in early-stage nasopharyngeal carcinoma patients.	Kam MK,Leung SF,Zee B,Chau RM,Suen JJ,Mo F,Lai M,Ho R,Cheung KY,Yu BK,Chiu	10.1200/JCO.2007.11.5501	https://pubmed.ncbi.nlm.nih.gov/17971582/

Restricted Information and Basic Personal Data

Title	Authors	DOI	PMID URL
	SK,Choi PH,Teo PM,Kwan WH,Chan AT		
Role of intensity-modulated radiotherapy in reducing toxicity in dose escalation for localized prostate cancer.	Al-Mamgani A,Heemsbergen WD,Peeters ST,Lebesque JV	10.1016/j.ijrobp.2008.04.063	https://pubmed.ncbi.nlm.nih.gov/18718725/
Systematic review and meta-analyses of intensity-modulated radiation therapy versus conventional two-dimensional and/or or three-dimensional radiotherapy in curative-intent management of head and neck squamous cell carcinoma.	Gupta T,Kannan S,Ghosh-Laskar S,Agarwal JP	10.1371/journal.pone.0200137	https://pubmed.ncbi.nlm.nih.gov/29979726/
[A randomized study of intensity-modulated radiation therapy versus three dimensional conformal radiation therapy for pelvic radiation in patients of post-operative treatment with gynecologic malignant tumor].	Ni J,Yin ZM,Yuan SH,Liu NF,Li L,Xu XX,Lou HM	10.3760/cma.j.issn.0529-567X.2017.03.006	https://pubmed.ncbi.nlm.nih.gov/28355688/
Postoperative radiotherapy with intensity-modulated radiation therapy versus 3-dimensional conformal radiotherapy in early breast cancer: A randomized clinical trial of KROG 15-03.	Choi KH,Ahn SJ,Jeong JU,Yu M,Kim JH,Jeong BK,Lee JH,Kim SH,Lee JH	10.1016/j.radonc.2020.09.043	https://pubmed.ncbi.nlm.nih.gov/32980384/
Accelerated partial breast irradiation using intensity-modulated radiotherapy versus whole breast irradiation: 5-year survival analysis of a phase 3 randomised controlled trial.	Livi L,Meattini I,Marrazzo L,Simontacchi G,Pallotta S,Saieva C,Paiar F,Scotti V,De Luca Cardillo C,Bastiani P,Orzalesi L,Casella D,Sanchez L,Nori J,Fambrini M,Bianchi S	10.1016/j.ejca.2014.12.013	https://pubmed.ncbi.nlm.nih.gov/25605582/
A prospective, randomized study comparing outcomes and toxicities of intensity-modulated radiotherapy vs. conventional two-dimensional radiotherapy for the treatment of nasopharyngeal carcinoma.	Peng G,Wang T,Yang KY,Zhang S,Zhang T,Li Q,Han J,Wu G	10.1016/j.radonc.2012.08.013	https://pubmed.ncbi.nlm.nih.gov/22995588/
Quality of life after simultaneously integrated boost with intensity-modulated versus conventional radiotherapy with sequential boost for adjuvant treatment of breast cancer: 2-year results of the multicenter randomized IMRT-MC2 trial.	Forster T,Hommertgen A,HÃ¤fner MF,Arians N,KÃ¶nig L,Harrabi SB,Schlampp I,KÃ¶hler C,Meixner E,Heinrich V,Weidner N,HÃ¼sing J,Sohn C,Heil J,Golatta M,Hof H,Krug D,Debus J,HÃ¶rner-Rieber J	10.1016/j.radonc.2021.08.019	https://pubmed.ncbi.nlm.nih.gov/34480960/
Quality of Life: A Prospective Randomized Trial of Palliative Volumetric Arc Therapy Versus 3-Dimensional Conventional Radiation Therapy.	Wong P,Lambert L,Thanomsack P,Coulombe G,Lambert C,Charpentier AM,Barkati M,Fortin I,Lafontaine J,Roberge D	10.1016/j.ijrobp.2020.11.061	https://pubmed.ncbi.nlm.nih.gov/33259935/
Postoperative adjuvant IMRT for patients with HCC and portal vein tumor thrombus: An open-label randomized controlled trial.	Sun J,Yang L,Shi J,Liu C,Zhang X,Chai Z,Lau WY,Meng Y,Cheng SQ	10.1016/j.radonc.2019.05.006	https://pubmed.ncbi.nlm.nih.gov/31176205/
Parotid-sparing intensity modulated versus conventional radiotherapy in head and neck cancer (PARSPORT): a phase 3 multicentre randomised controlled trial.	Nutting CM,Morden JP,Harrington KJ,Urbano TG,Bhide SA,Clark C,Miles EA,Miah AB,Newbold K,Tanay M,Adab F,Jefferies SJ,Scrase	10.1016/S1470-2045(10)70290-4	https://pubmed.ncbi.nlm.nih.gov/21236730/

Restricted Information and Basic Personal Data

Title	Authors	DOI	PMID URL
	C,Yap BK,A'Hern RP,Sydenham MA,Emson M,Hall E		
A multicenter randomized trial of breast intensity-modulated radiation therapy to reduce acute radiation dermatitis.	Pignol JP,Olivotto I,Rakovitch E,Gardner S,Sixel K,Beckham W,Vu TT,Truong P,Ackerman I,Paszat L	10.1200/JCO.2007.15.2488	https://pubmed.ncbi.nlm.nih.gov/18285602/
Intensity-modulated radiation therapy versus three-dimensional conformal radiotherapy in head and neck squamous cell carcinoma: long-term and mature outcomes of a prospective randomized trial.	Gupta T,Sinha S,Ghosh-Laskar S,Budrukkar A,Mummudi N,Swain M,Phurailatpam R,Prabhash K,Agarwal JP	10.1186/s13014-020-01666-5	https://pubmed.ncbi.nlm.nih.gov/32938468/
Quality-of-life (QOL) outcomes in patients with head and neck squamous cell carcinoma (HNSCC) treated with intensity-modulated radiation therapy (IMRT) compared to three-dimensional conformal radiotherapy (3D-CRT): evidence from a prospective randomized study.	Rathod S,Gupta T,Ghosh-Laskar S,Murthy V,Budrukkar A,Agarwal J	10.1016/j.oraloncology.2013.02.013	https://pubmed.ncbi.nlm.nih.gov/23562564/
Prospective randomized controlled trial to compare 3-dimensional conformal radiotherapy to intensity-modulated radiotherapy in head and neck squamous cell carcinoma: Long-term results.	Ghosh-Laskar S,Yathiraj PH,Dutta D,Rangarajan V,Purandare N,Gupta T,Budrukkar A,Murthy V,Kannan S,Agarwal JP	10.1002/hed.24263	https://pubmed.ncbi.nlm.nih.gov/26561342/
Intensity Modulated Radiation Therapy (IMRT) With Simultaneously Integrated Boost Shortens Treatment Time and Is Noninferior to Conventional Radiation Therapy Followed by Sequential Boost in Adjuvant Breast Cancer Treatment: Results of a Large Randomized Phase III Trial (IMRT-MC2 Trial).	HÃ¶rner-Rieber J,Forster T,Hommertgen A,Haefner MF,Arians N,KÃ¶nig L,Harrabi SB,Schlampp I,Weykamp F,Lischalk JW,Heinrich V,Weidner N,HÃ¼sing J,Sohn C,Heil J,Hof H,Krug D,Debus J	10.1016/j.ijrobp.2020.12.005	https://pubmed.ncbi.nlm.nih.gov/33321192/
A Prospective Randomized Study of Intensity-Modulated Radiation Therapy Versus Three-Dimensional Conformal Radiation Therapy With Concurrent Chemotherapy in Locally Advanced Carcinoma Cervix.	Sharma N,Gupta M,Joseph D,Gupta S,Pasricha R,Ahuja R,Krishnan AS,T S A,Raut S,Sikdar D	10.7759/cureus.21000	https://pubmed.ncbi.nlm.nih.gov/35154974/
Efficacy and Safety of Locoregional Radiotherapy With Chemotherapy vs Chemotherapy Alone in De Novo Metastatic Nasopharyngeal Carcinoma: A Multicenter Phase 3 Randomized Clinical Trial.	You R,Liu YP,Huang PY,Zou X,Sun R,He YX,Wu YS,Shen GP,Zhang HD,Duan CY,Tan SH,Cao JY,Li JB,Xie YL,Zhang YN,Wang ZQ,Yang Q,Lin M,Jiang R,Zhang MX,Hua YJ,Tang LQ,Zhuang AH,Chen QY,Guo L,Mo HY,Chen Y,Mai HQ,Ling L,Liu Q,Chua MLK,Chen MY	10.1001/jamaoncol.2020.1808	https://pubmed.ncbi.nlm.nih.gov/32701129/
A randomized study for dosimetric assessment and clinical impact of bone marrow sparing intensity-modulated radiation therapy versus 3-dimensional conformal radiation therapy on hematological and gastrointestinal toxicities in cervical cancer.	Kapoor AR,Bhalavat RL,Chandra M,Pareek V,Moosa Z,Markana S,Nandakumar P,Bauskar P,Shincy NV	10.4103/jcrt.JCRT_1242_20	https://pubmed.ncbi.nlm.nih.gov/36412399/

Restricted Information and Basic Personal Data

Title	Authors	DOI	PMID URL
Intensity-modulated radiation therapy for head and neck cancer: systematic review and meta-analysis.	Marta GN,Silva V,de Andrade Carvalho H,de Arruda FF,Hanna SA,Gadia R,da Silva JL,Correa SF,Vita Abreu CE,Riera R	10.1016/j.radonc.2013.11.010	https://pubmed.ncbi.nlm.nih.gov/24332675/
Comparison of dosimetric parameters and acute toxicity of intensity-modulated and three-dimensional radiotherapy in patients with cervix carcinoma: A randomized prospective study.	Naik A,Gurjar OP,Gupta KL,Singh K,Nag P,Bhandari V	10.1016/j.canrad.2016.05.011	https://pubmed.ncbi.nlm.nih.gov/27368915/
Palliative Radiation for Advanced Central Lung Tumors With Intentional Avoidance of the Esophagus (PROACTIVE): A Phase 3 Randomized Clinical Trial.	Louie AV,Granton PV,Fairchild A,Bezjak A,Gopaul D,Mulroy L,Brade A,Warner A,Debenham B,Bowes D,Kuk J,Sun A,Hoover D,Rodrigues GB,Palma DA	10.1001/jamaoncol.2021.7664	https://pubmed.ncbi.nlm.nih.gov/35201290/
A randomized, controlled, multicenter study comparing intensity-modulated radiotherapy plus concurrent chemotherapy with chemotherapy alone in gastric cancer patients with D2 resection.	Zhu WG,Xua DF,Pu J,Zong CD,Li T,Tao GZ,Ji FZ,Zhou XL,Han JH,Wang CS,Yu CH,Yi JG,Su XL,Ding JX	10.1016/j.radonc.2012.08.024	https://pubmed.ncbi.nlm.nih.gov/22985776/
Intensity-modulated radiotherapy reduces toxicity with similar biochemical control compared with 3-dimensional conformal radiotherapy for prostate cancer: A randomized clinical trial.	Viani GA,Viana BS,Martin JE,Rossi BT,Zuliani G,Stefano EJ	10.1002/cncr.29983	https://pubmed.ncbi.nlm.nih.gov/27028170/
Quality of Life and Radiation-induced Late Toxicity Following Intensity-modulated Versus Three-dimensional Conformal Radiotherapy for Patients with Spinal Bone Metastases: Results of a Randomized Trial.	Sprave T,Verma V,FÃ¶rster R,Schlampp I,Hees K,Bruckner T,Bostel T,El Shafie R,Nicolay NH,Debus J,Rief H	10.21873/anticanres.12813	https://pubmed.ncbi.nlm.nih.gov/30061275/

Restricted Information and Basic Personal Data
